# Functional Characterization of Floral Gene Network Reveals a Critical FT1–AP1 Interaction in Flowering Regulation in Longan

**DOI:** 10.3390/plants15010106

**Published:** 2025-12-30

**Authors:** Yuru Tang, Yating Xu, Haoming Mao, Yawen Xu, Jianling Pan, Shaoquan Zheng, Guochun Zhao, Wenshun Hu, Ray Ming

**Affiliations:** 1 Fujian Provincial Key Laboratory of Haixia Applied Plant Systems Biology, Center for Genomics and Biotechnology, Fujian Agriculture and Forestry University, Fuzhou 350002, China; 2 Key Laboratory of Genetics, Breeding and Multiple Utilization of Corps, Ministry of Education, Fujian Agriculture and Forestry University, Fuzhou 350002, China; 3 Fujian Breeding Engineering Technology Research Center for Longan and Loquat, Fruit Research Institute, Fujian Academy of Agricultural Sciences, Fuzhou 350013, China

**Keywords:** *DlAP1*, *DlFD*, *DlFT1*, *DlFT2*, floral induction, longan, protein interaction

## Abstract

Longan (*Dimocarpus longan* Lour.) is a commercially valuable tropical fruit crop that contains two antagonistic *FLOWERING LOCUS T* (*FT*) homologs involved in regulating flowering time. However, how these *FT* genes interact with flowering regulators *FLOWERING LOCUS D* (*FD*) and *APETALA1* (*AP1*) remains unknown. Four flowering-related genes in longan, *DlFT1*, *DlFT2*, *DlAP1* and *DlFD*, were successfully isolated. Expression profiling revealed that all four genes were expressed in leaves and buds across different stages of natural and KClO_3_-induced floral bud differentiation. Functional characterization through heterologous overexpression in *Arabidopsis thaliana* showed that *DlAP1* significantly promotes early flowering under long-day conditions and induced morphological changes in floral organs and leaves. In contrast, *DlFD* overexpression had no effect on flowering time. Subcellular localization assays revealed that DlFT1 and DlFT2 localized to both the nucleus and the plasma membrane, while DlAP1 and DlFD localized exclusively to the nucleus. Yeast two-hybrid and bimolecular fluorescence complementation (BiFC) analyses revealed a novel regulatory node: DlFT1 directly interacts with DlAP1, a finding that expands the classical FT-FD-AP1 flowering model. Additionally, DlFD interacts more strongly with DlFT1 than with DlFT2, whereas DlFT1 only interacts with DlAP1, but not DlFT2. These results demonstrate that DlFT1 promotes flowering not only via the conserved FD-dependent pathway but also through direct association with AP1. These findings advance our understanding of the regulatory mechanisms of flowering in longan and provide valuable insights into flowering pathways of perennial woody species.

## 1. Introduction

Flowering in plants is an important physiological process involving the transition from vegetative growth to reproductive growth, which is precisely regulated by endogenous and exogenous signals. Flowering induction, the critical stage initiating reproductive growth, forms the basis of plant flowering initiation. Deciphering its regulatory network plays an important role in plant flowering improvement and plant breeding. Currently, a complex and sophisticated flowering regulatory network has been established in the model plant *A*. *thaliana*, and *FT* is a central integrator gene in the flowering regulatory network, integrating multiple endogenous and exogenous signals. It is a member of the phosphatidylethanolamine-binding protein (PEBP) family with a conserved PEBP structural domain and is a small mobile protein [1]. *FT* is produced in leaf phloem companion cells and then transported through the phloem to the shoot apical meristem (SAM), where it interacts with *FD* to form the florigen activation complex (FAC), which activates the expression of the floral meristem gene *AP1*, thereby initiating the flowering process [2]. *FD*, a member of the basic leucine zipper (bZIP) transcription factor family [3], is predominantly expressed in the shoot apical meristem (SAM) [2], and is the most critical flowering cofactor in the flowering regulatory network.

*AP1* is a floral meristem organization signature gene, and a floral organ morphology signature gene, which is also associated with sepal and petal development [4,5]. *FT*, *FD*, *AP1*, *TERMINAL FLOWER 1* (*TFL1*), and *LEAFY* (*LFY*) interact to coordinately regulate flower formation [6]. Recent studies have further elucidated the mechanistic details of this regulatory network. In Arabidopsis, the interaction between FT and FD is mediated by 14-3-3 proteins, forming a ternary complex that promotes *AP1* expression and drives floral organ formation [7]. Additionally, research has revealed that FD within the FAC not only binds FT, but also cooperates with other transcription factors such as SQUAMOSA PROMOTER BINDING PROTEIN-LIKEs (SPLs) to coordinately regulate the expression of *AP1* and *LFY*, further advancing floral organ development [8]. These findings underscore the central roles of *FT*, *FD*, and *AP1* in the flowering regulatory network. As a mobile signaling molecule, FT interacts with FD to assemble the FAC, which activates AP1 transcription and ultimately triggers the transition from the vegetative growth phase to the reproductive growth phase in plants. The ABC model (later expanded into the ABCDE model) is a widely recognized model for regulating the differentiation and development of plant floral organs, which suggests that genes of classes A, B, C, D, and E individually or in combination control the determination and development of floral organs in each whorl of the plant [9,10]. Among them, class A genes determine sepal and petal development. *AP1* is also a typical class A gene encoding a MADS-box protein that coordinates floral development by integrating growth, pattern formation, and hormonal pathways [11].

The key flowering genes *FT*, *FD*, and *AP1* have been well-characterized in Arabidopsis, and their interactions have been elucidated as central components of the flowering regulatory network, providing important implications for related studies in other species. Longan is a commercially important fruit tree in the tropics and subtropics. Crucially, its flowering can be reliably induced off-season by potassium chlorate (KClO_3_) application, a key technology for extending fruit production periods [12]. However, the regulatory mechanisms of its flowering network have not been fully resolved. Currently, the functions of two homologous genes of Arabidopsis *FT* in longan, *DlFT1* and *DlFT2*, have been analyzed, and *DlFT1* and *DlFT2* have opposing roles in flowering regulation. Overexpression of *DlFT1* in Arabidopsis accelerates flowering, indicating its potential role as a flowering promoter, while overexpression of *DlFT2* leads to delayed flowering and defects in floral organ development, suggesting that it may function as a flowering repressor [13,14,15]. However, the functions of *DlAP1* and *DlFD* remain poorly characterized, and their interactions with other flowering regulators are not well-understood. An in-depth investigation of their functions and relationships with the roles of *DlFT1* and *DlFT2* is crucial for revealing the molecular mechanisms of flowering regulation in longan.

The aim of this study was to clone the key flowering genes *DlAP1* and *DlFD* in longan and to explore their functions and their interactions with *DlFT1* and *DlFT2*. We found that ectopic expression of *DlAP1* in Arabidopsis significantly promoted flowering and altered floral organ development. Subcellular localization showed that all these genes were localized to the nucleus or nuclear membrane. Notably, in protein–protein interactions, the two FT proteins that showed differences in flowering regulation still exhibited differences. DlAP1 interacts with flowering promoter DlFT1 but not with the flowering repressor DlFT2. Similarly, DlFD binds to both DlFT1 and DlFT2, but yeast two-hybrid assays revealed that its interaction with DlFT1 is stronger than with DlFT2, suggesting a selective partnership in flowering regulation. These findings provide new perspectives for the study of the flowering regulatory network in longan.

## 2. Results

### 2.1. Sequence Comparison and Phylogenetic Analysis of DlAP1 and DlFD

We obtained the CDS sequences of *DlAP1* and *DlFD* by RACE cloning. The length of *DlAP1* is 726 bp, encoding a protein containing 241 amino acids; the length of *DlFD* is 909 bp, encoding a protein containing 302 amino acids. Conserved domain analysis using NCBI Conserved Domain Search Service (CD-Search) revealed that DlAP1 contains a MADS-box domain [16], which is highly similar to proteins AP1 and FRUITFULL (FUL) in the Arabidopsis MADS-box family. We further obtained the homologous proteins of DlAP1 from GenBank public database and SapBase (Sapinaceae Genomic DataBase) [17], and then performed sequence comparison and phylogenetic tree construction.

**Figure 1:**
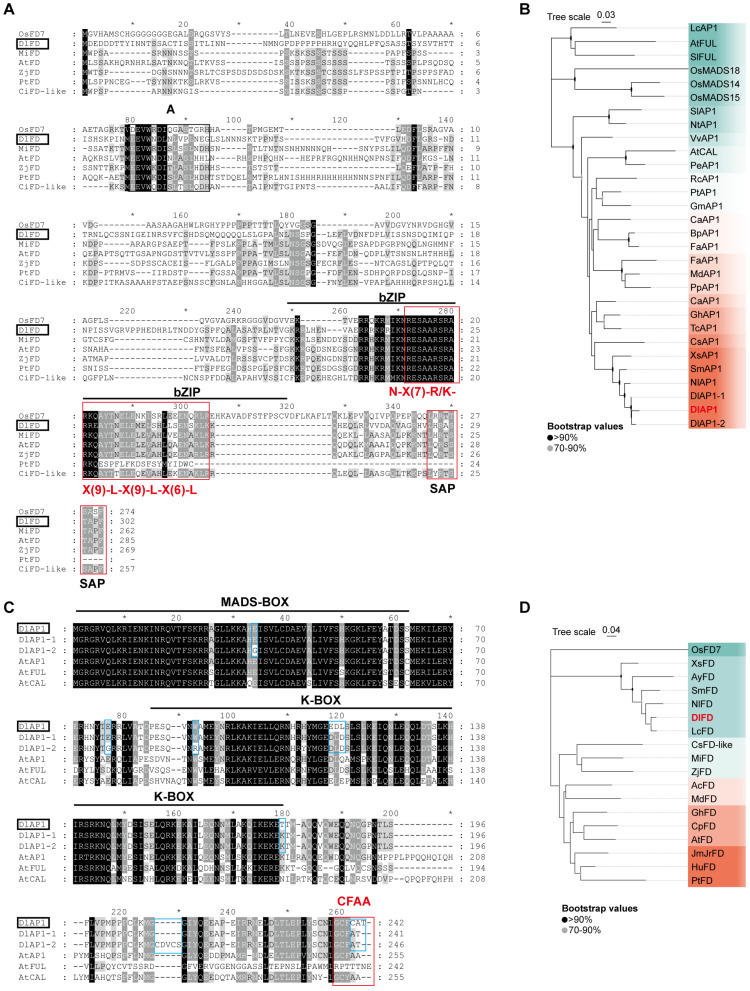
Sequence comparison and phylogenetic tree construction of DlAP1 and DlFD with other homologous proteins. (**A**) Comparison of DlAP1 homologous proteins with black background indicating fully conserved amino acid residues and different shades of gray representing amino acids with different degrees of conservation. Black horizontal lines indicate MADS-box and K-box structural domains, red boxes indicate Arabidopsis C-terminal ‘*CFAA*’ motifs. (**B**) Phylogenetic tree of DlAP1 and homologous proteins from Sapindaceae (e.g., longan, lychee), rice, maize, and Arabidopsis AP1. (**C**) Comparison of DlFD homologous proteins, black horizontal lines indicate the bZIP structural domains, black filament boxes indicate ‘A’ and ‘SAP’ motifs, red boxes indicate ‘N-X(7)-R/K-X(9)-L-X(9)-L-X(6)-L-X(6)-L’ motifs, and blue boxes highlight amino acid differences between DlAP1 and its variants (DlAP1-1/2). An asterisk (*) in (**A**,**C**) indicates positions with fully conserved amino acid residues across all aligned sequences. (**D**) Phylogenetic tree of DlFD and homologous proteins of the Sapindaceae family and the homologous proteins of Mango, Maize, and Arabidopsis FD. The first two letters of each protein represent the species scientific name, *Ac*, *Actinidia chinensis*; *At*, *Arabidopsis thaliana*; *Ay*, *Acer yangbiense*; *Bp*, *Betula platyphylla*; *Ca*, *Corylus avellana*; *Cp*, *Carica papaya*; *Cs*, *Citrus sinensis*; *Dl*, *Dimocarpus longan*; *Fa*, *Fragaria xananassa*; *Gh*, *Gossypium hirsutum*; *Gm*, *Glycine max*; *Hu*, *Herrania umbratica*; *JmJr*, *Juglans microcarpa × Juglans regia*; *Lc*, *Litchi chinensis*; *Md*, *Malus domestica*; *Mi*, *Mangifera indica*; *Nl*, *Nephelium lappaceum*; *Nt*, *Nicotiana tabacum*; *Os*, *Oryza sativa*; *Pe*, *Passiflora edulis*; *Pp*, *Prunus persica*; *Pt*, *Populus trichocarpa*; *Rc*, *Ricinus communis*; *Sm*, *Sapindus mukorossi*; *Sl*, *Solanum lycopersicum*; *Tc*, *Theobroma cacao*; *Vv*, *Vitis vinifera*; *Xs*, *Xanthoceras sorbifolium*; *Zj*, *Ziziphus jujuba*.

The N-terminal MADS-box domain of the DlAP1 protein was highly conserved with its homologous proteins; in addition, it also contained a moderately conserved K-box structural domain and the C-terminal AP1 motif ‘CFAA’ [18] (Figure 1A). The MADS-box structural domain of DlAP1 conforms to the typeⅡ MADS-box gene signature (i.e., the DlAP1 protein is a MIKC-type MADS-domain protein) [19]. Phylogenetic analyses further showed that DlAP1 had higher similarity with homologous proteins in Sapindaceae, with global similarity to *Nephelium lappaceum* AP1 (NlAP1, 92.9%), *Sapindus mukorossi* AP1 (SmAP1, 90.6%), and *Xanthoceras sorbifolium* AP1 (XsAP1, 83.5%), and thus DlAP1 clustered with Sapindaceae family members in the phylogenetic tree (Figure 1B). The similarity of DlAP1 with AP1, CAULIFLOWER (CAL), and FUL was 59.8%, 51.2%, and 24.3%, respectively.

Similar to DlAP1, the DlFD protein contains two conserved motifs, A and SAP, with a conserved basic leucine zipper (bZIP) structure, characteristic of bZIP transcription factors. In the sequence alignment, the red box highlights the conserved motif typical of this structure [N-X(7)-R/K-X(9)-L-X(9)-L-X(6)-L-X(6)-L] (Figure 1C). Phylogenetic analysis showed that DlFD shared the highest sequence homology with FD homologs from *Litchi chinensis* FD (LcFD, 95.2%), *N. lappaceum* FD (NlFD, 88.4%), *S. mukorossi* FD (SmFD, 85.7%), *X. sorbifolium* FD (XsFD, 76.3%), and *Acer yangbiense* FD (AyFD, 76.3%). Among these, DlFD clustered most closely with SmFD in the same clade of the phylogenetic tree (Figure 1D).

### 2.2. Expression Pattern of DlAP1, DlFD and DlFT1/2 in Longan Flowering

To investigate the molecular mechanisms underlying floral initiation under both natural and KClO_3_-induced off-season flowering, we collected bud and leaf tissues prior to the onset of floral bud morphological differentiation. Sampling covered a 50-day period (six stages) under natural growth condition and 30-day period (three stages) under KClO_3_-induced off-season flowering. Using RT-qPCR, we analyzed the expression patterns of *DlFT1*, *DlFT2*, *DlAP1*, and *DlFD*, revealing distinct tissue-specific and temporal regulation during the pre-floral transition phase.

**Figure 2:**
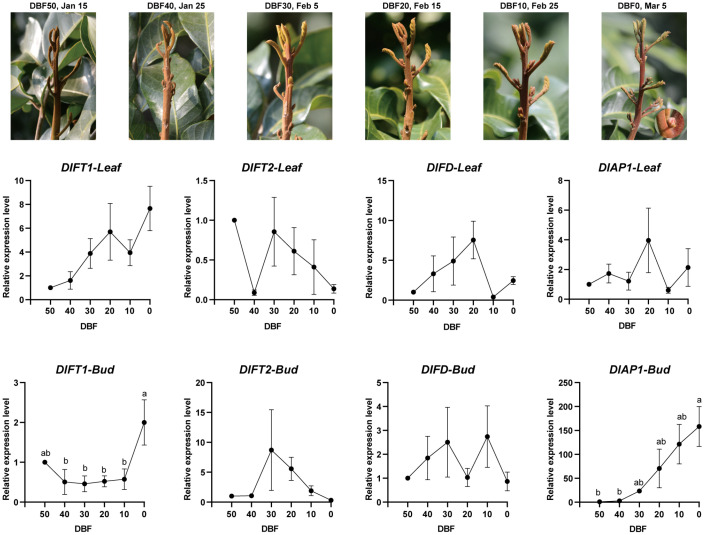
Morphological development and expression profiles of *DlFT1*, *DlFT2*, *DlFD*, and *DlAP1* during natural floral transition in longan. (**Top**): Representative bud images at six developmental stages spanning 50 days before floral bud differentiation (50 DBF to 0 DBF). (**Bottom**): Gene expression patterns in leaf (solid lines) and bud (dashed lines) tissues quantified by RT-qPCR using 2^−ΔΔCT^ normalization. Data represent mean ± SD from three biological replicates. For data across different time points, one-way ANOVA was first performed, followed by the Tukey–Kramer post hoc test for multiple comparisons if a significant difference was detected. Groups not sharing the same letter were statistically different at the 5% significance level.

Under natural floral induction conditions (Figure 2), *DlFT1* exhibited contrasting tissue-specific expression: in leaves, it was gradually upregulated throughout the pre-floral phase, while in buds, its expression remained stable until 10 days before flowering (10 DBF), followed by a sharp increase from 10 DBF to 0 DBF. *DlFT2* exhibited a biphasic pattern in leaves, with a sharp decline reaching the minimum at 40 DBF, partial recovery at 30 DBF and subsequent attenuation. In buds, *DlFT2* expression was undetectable until 30 DBF, peaking at this critical transition point then declining. *DlFD* also displayed divergent patterns: in leaves, it peaked at 20 DBF and was sharply downregulated at 10 DBF, whereas in buds, its expression gradually increased until 30 DBF, dipped slightly at 20 DBF, and peaked again at 10 DBF. Notably, *DlAP1* expression in leaves remained mostly stable, with a distinct transient peak at 20 DBF, whereas in buds, it showed sustained upregulation through the pre-floral phase.

**Figure 3:**
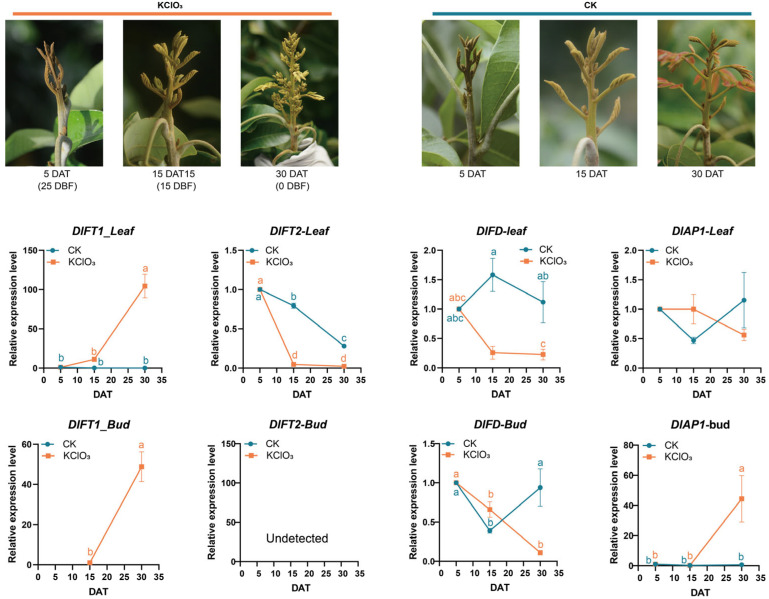
Morphological response and expression profiles of DlFT1, DlFT2, DlFD, and DlAP1 in the KClO_3_-induced flowering of longan trees. (**Top**): Representative bud images at 5, 15, and 30 days after treatment (DAT), that is 25, 15 and 0 days before flowering (DBF). (**Bottom**): Gene expression patterns in leaf (solid lines) and bud (dashed lines) tissues quantified by RT-qPCR using 2^−ΔΔCT^ normalization. Data represent mean ± SD from three independent biological replicates. The transcripts of *DlFT1* (bud tissue, CK group) and *DlFT2* (bud tissue, CK and KClO_3_ groups) were below the detection limit of RT-qPCR. Following a significant one-way ANOVA, group means were compared by the Tukey–Kramer test. Different letters assigned to the groups denote statistically significant differences at *p* < 0.05.

KClO_3_ treatment fundamentally altered these expression dynamics of flowering related genes in longan (Figure 3). Following KClO_3_ treatment, *DlFT1* expression was significantly induced in both tissues, in stark contrast to the control group. In leaves, *DlFT1* showed a progressive upregulation, inversely mirroring the expression trend seen in untreated plants. In buds, *DlFT1* was induced de novo starting at 15 DAT. *DlFT2* exhibited a differential response: its leaf expression dropped sharply to a minimum at 15 DAT (unlike the gradual decline in controls), while its expression in bud remained undetected in both the treated and control groups.

*DlFD* expression displayed a treatment-specific inversion. In KClO_3_-treated leaves, it peaked early at 5 DAT and was subsequently suppressed, whereas in control leaves, it peaked later at 15 DAT; in buds, KClO_3_ treatments led to progressive downregulation, in contrast to fluctuating patterns observed in the controls. Notably, *DlAP1* expression in KClO_3_-treated buds became tightly synchronized with *DlFT1*, both showing continuous upregulation.

These results demonstrate that KClO_3_ treatment reprogrammed the transcriptional hierarchy of floral regulators, synchronizing *DlAP1* induction with *DlFT1* upregulation while uncoupling *DlFD* from its natural expression rhythm. The observed tissue-specific responses suggest that *DlFT1* acted as the primary floral promoter during KClO_3_-induced flowering, potentially through coordinated activation with *DlAP1* in meristematic tissues.

### 2.3. Heterologous Overexpression of DlAP1 and DlFD

**Figure 4:**
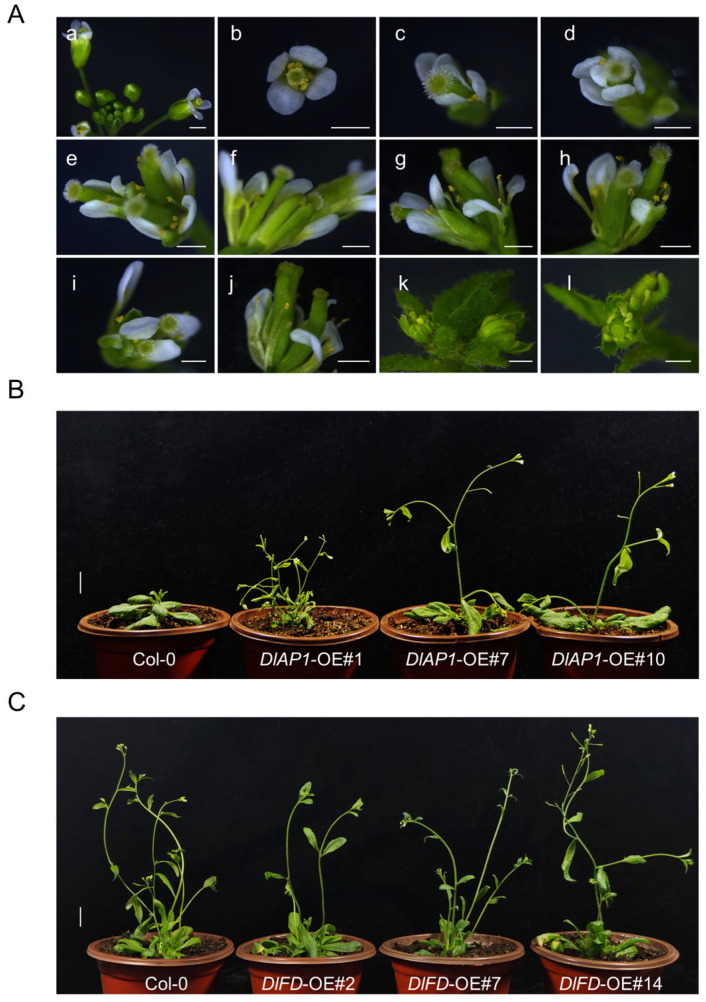
Overexpression of *DlAP1* and *DlFD* in Col-0 Arabidopsis. (**A**) Phenotypic Comparison of Col-0 and *DlAP1*-OE#1 in Flowering Organs. (**a**,**b**) Col-0, (**c**,**d**) Increased petals, (**e**,**f**) Conversion of terminal buds to terminal flowers, (**g**,**h**) Conversion of inflorescence branches to solitary flowers, (**i**,**j**) Two pistils, (**k**,**l**) Abnormal petal development. Scale bar = 1 µm. (**B**) Overexpression of *DlAP1* in Col-0 prompted earlier flowering. (**C**) Overexpression of *DlFD* in Col-0 did not result in a significant phenotype in comparison with the wild type. Scale bar = 1 cm in (**B**,**C**).

**Figure 5:**
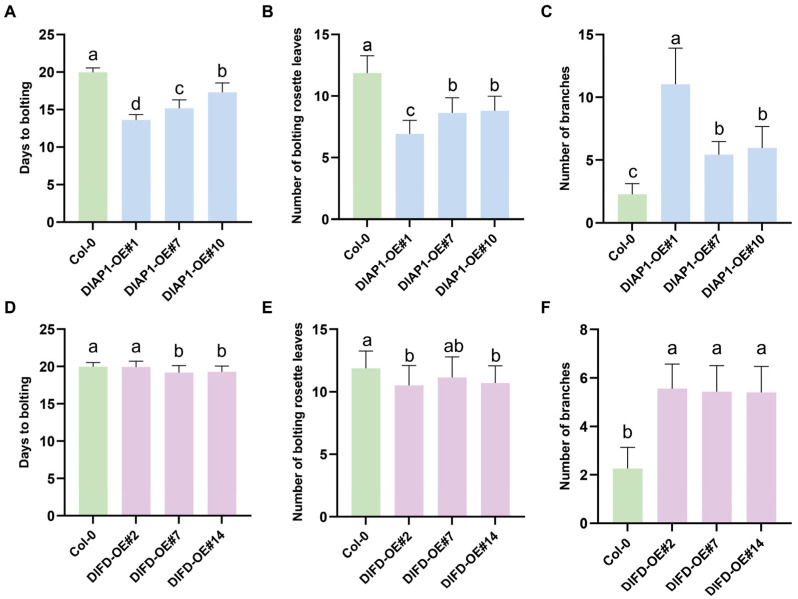
Phenotypic statistics of *DlAP1* versus *DlFD* overexpression in Arabidopsis under long day. (**A**) Flowering phenotypes of Col-0 and *DlAP1*-OE#1/#7/10 plants. (**B**) The numbers of rosette leaves in Col-0 and *DlAP1*-OE#1/#7/10 upon bolting. (**C**) The total number of branches in Col-0 and *DlAP1*-OE#1/#7/10. (**D**) Flowering phenotypes of Col-0 and *DlFD*-OE#2/#7/#14 upon bolting. (**E**) The numbers of rosette leaves in Col-0 and *DlFD*-OE#2/#7/#14 upon bolting. (**F**) The total number of branches in Col-0 and *DlFD*-OE#2/#7/#14. Multiple comparisons were performed using the Tukey–Kramer method. Groups not sharing the same letter indicate statistically significant differences (*p* < 0.05).

To investigate the functions of *DlAP1* and *DlFD*, transgenic lines of Arabidopsis overexpressing these genes were generated, and their flowering phenotypes were analyzed. From the T1 generation of *DlAP1*-overexpressing lines, three lines displaying distinct flowering phenotypes were selected for screening homozygous lines. Under long day conditions, T3 generation plants flowered significantly earlier than Col-0 plants, with flowering times advanced by approximately 6, 5, and 3 days, respectively. Additionally, the number of rosette leaves at bolting was significantly reduced, while branch numbers increased, particularly in the *DlAP1*-OE#1 line (Figure 4B and Figure 5A–C).

Notably, compared to Col-0 and *DlAP1*-OE#7/#10, the *DlAP1*-OE#1 line exhibited a dwarf phenotype, a shorter flowering duration, conversion of inflorescences into solitary flowers, and altered floral organ morphology (Figure 4A,B). In this line, 41.5% plants (*n* = 53) produced two pistil flower, and 45% of plants (*n* = 50) developed flowers with 5–7 petals, whereas stamen and sepal numbers remained largely unchanged. In some cases, leaves from flowering stem arose from the axillary position and formed terminal flowers (Figure 4A(g,h)). Most *DlAP1*-OE#1 plants developed young spoon-shaped rosette leaves, although this phenotype diminished as leaves matured.

In contrast, no significant changes in flowering time were observed in the *DlFD*-OE T1 generations. To further assess its role, RT-qPCR was used to measure transgenic expression levels, and three lines (*DlFD*-OE#2/#7/#14) with the highest expression were selected for homozygous screening. However, even in the T3 generation, these lines exhibited no notable function in flowering time and rosette leaf number under long-day conditions (Figure 4 and Figure 5D–F).

### 2.4. Interaction Analysis of DlFT1 and DlFT2 with DlAP1 and DlFD

In the model plant Arabidopsis, FT functions as a core component of the florigen and forms a complex with FD and AP1 to jointly regulate the flowering transition [1,2]. However, it remains unclear whether FT homologs regulate flowering through a similar network of protein–protein interactions in perennial woody plants such as longan.

**Figure 6:**
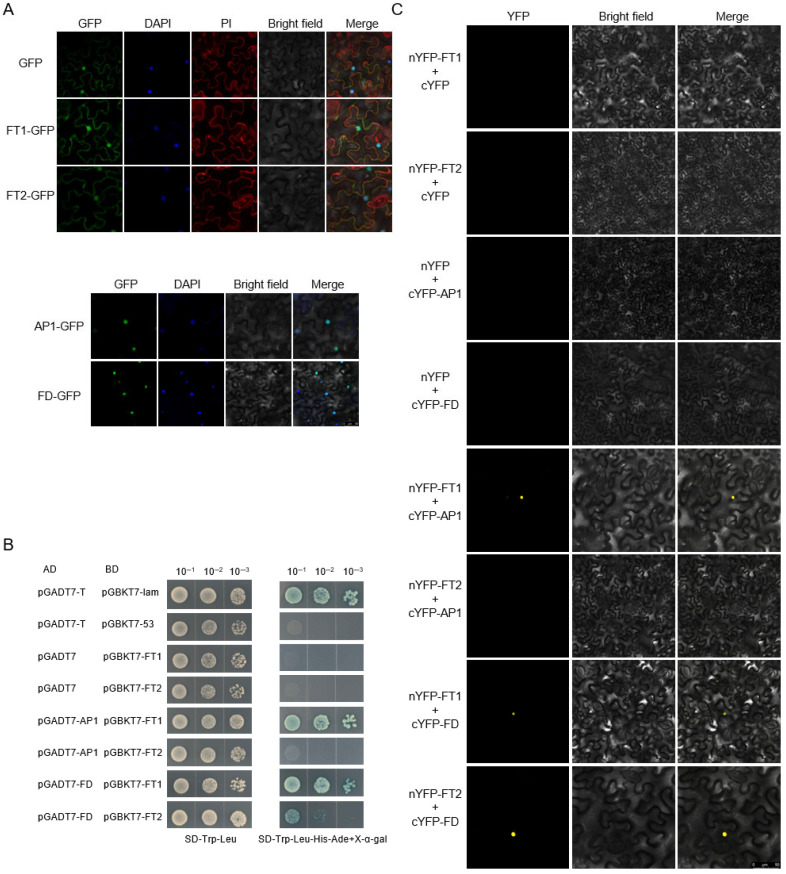
Subcellular localization and interaction analysis between proteins DlFT1/2 and DlAP1/DlFD. (**A**) Subcellular localization of DlFT1, DlFT2, DlAP1, and DlFD. (**B**) Yeast two-hybrid analysis of interactions between DlFT1/DlFT2 and DlAP1/DlFD. (**C**) BiFC analysis of interactions between DlFT1/DlFT2 and DlAP1/DlFD.

To investigate the subcellular localization of proteins DlFT1, DlFT2, DlAP1, and DlFD in vivo, we transiently expressed their green fluorescent protein (GFP) fusion constructs in Ben’s tobacco (*Nicotiana benthamiana*) cells. DAPI and PI staining were used as nuclear and plasma membrane markers, respectively. *DlFT1-GFP* and *DlFT2-GFP* signals were present in both the nucleus and plasma membrane, whereas *DlAP1-GFP* and *DlFD-GFP* were exclusively localized in the nucleus (Figure 6A).

Subsequently, yeast two-hybrid assays using a nuclear expression system were performed to investigate the interaction relationship between proteins DlAP1/DlFD and the functionally distinct protein DlFT1 and DlFT2. All transformants grew normally on double-dropout medium (SD/-Leu/-Trp), confirming successful plasmid transformation and ruling out toxicity or autoactivation. Strong interactions were detected between DlFT1–DlAP1 and DlFT1–DlFD. A week interaction was detected between DlFT2 and DlFD, while no interaction was observed between DlFT2 and DlAP1 on the quadruple dropout medium (SD/-Leu/-Trp/-His/-Ade+x-α-gal) (Figure 6B).

To further validate these interactions, bimolecular fluorescence complementation (BiFC) assays were conducted using the tobacco transient transformation system. DlFT1 and DlFT2 were fused to the YFP (YN), while DlAP1 and DlFD were fused to the C-terminal fragment (YC). All combinations were co-infiltrated into tobacco leaf cells, and YN/YC empty vector combinations served as negative controls. Consistent with the yeast two-hybrid results, strong YFP fluorescence signals were observed in the nuclei of tobacco cells for DlFT1/2–DlFD and DlFT1–DlAP1 interactions, no BiFC signal was observed for DlFT2-DlAP1. Negative controls showed no fluorescence (Figure 6C).

These results collectively indicate that the flowering-promoting protein DlFT1 interacts with both DlAP1 and DlFD, whereas the flowering-repressing protein DlFT2 interacts only with DlFD. Given that AP1–FT interaction has not been documented in Arabidopsis or other established model systems, we further examined its specificity by constructing Y2H vectors for AtFT and AtAP1 and assaying their interactions with DlAP1 and DlFT1 from longan. These comparative experiments aimed to establish whether this interaction represents a unique regulatory mechanism in longan. The results showed that DlFT1 not only interacts with its homolog DlAP1 but also with Arabidopsis AtAP1 (Figure S1). Furthermore, using the Arabidopsis AtFT–AtAP1 pair as a control, we also observed positive interaction signals for these two proteins. However, we also noted interaction specificity: in the reverse combination, longan DlAP1 failed to bind to Arabidopsis AtFT.

## 3. Discussion

In the flowering regulatory network, AP1 acts as both a floral meristem identity gene and a downstream target of the photoperiod pathway. Previous studies have established that in *Arabidopsis*, FT-FD complexes activate *AP1* expression [2,20], and AP1 in turn reinforces this pathway by upregulating *FT* expression [21]. Our study uncovered a novel protein–protein interaction among these flowering regulators in longan, and we demonstrated that DlAP1 or DlFD can physically associate with flowering promoter DlFT1 in the nucleus, forming a direct regulatory module (Figure 6C,D). In contrast, DlAP1 does not interact with flowering suppressor DlFT2, and DlFD showed a lack of interaction with DlFT2. To assess the evolutionary conservation of this interaction, we performed Y2H assays and found that AtFT can also interact with AtAP1 (Figure S1). This suggests that the physical interaction between florigenic FT and AP1 may be widespread in plants, whereas such interactions may be absent with repressive FT homologs, a pattern not previously reported. Furthermore, the observed interaction between DlFT1 and AtAP1 implies that this association is likely biologically relevant and not merely a longan-specific phenomenon. These findings extend beyond the classical “FT–FD–AP1” regulatory framework and point to unexplored potential for direct FT–AP1 interactions across plant species, indicating a more complex model of flowering regulation.

The direct interaction between DlFT1 and DlAP1 is further supported by their convergent spatiotemporal expression patterns. Under KClO_3_ treatment, the synchronized upregulation of DlFT1 and DlAP1 in buds correlates with their physical interaction, suggesting that their co-activation promotes floral transition. In contrast, the absence of DlFT2–DlAP1 binding aligns with the low bud expression of DlFT2 and its non-inductive role in flowering. We propose that DlFT2, as a specialized flowering suppressor, may represent an adaptive mechanism in longan, a woody perennial, enabling more flexible integration of environmental and developmental cues to fine-tune flowering timing. The promotive function of DlFT1, repressive function of DlFT2, and their differential nuclear interaction with DlAP1 reflect recurrent adaptations in perennial flowering networks. Similar regulatory innovations have been observed in other perennials: for instance, in *Malus domestica*, MdTFL1 directly inhibits MdFT1 to synchronize seasonal flowering [22], and in *Populus*, duplicated *FT* genes (*PtFT1*/*PtFT2*) have diverged to coordinate spring flowering and autumn growth cessation [23]. Thus, the functional divergence and distinct interaction patterns among FT homologs in longan likely represent an evolutionary strategy to integrate florigen signaling (*FT*) with floral meristem identity (*AP1*), facilitating reproductive transition under perennial growth constraints.

To explore the molecular basis of these interactions, we first analyzed the domain architectures of DlAP1 and DlFD. DlAP1 contains an N-terminal MADS-box domain, a central K-box region likely involved in protein–protein dimerization, and a C-terminal region featuring the Arabidopsis AP1-like ‘CFAA’ motif while lacking the FUL-like ‘LPPWML’ motif [18,24,25] (Figure 1A). The DlFD protein contains two conserved motifs, ‘A’ and ‘SAP’ (Figure 1C), which may regulate the transcriptional activity of DlFD through protein interactions, such as recruiting LW-specific coactivators or responding to environmental signals. DlFD contains a conserved bZIP region that may regulate the expression of downstream related genes by forming homo- or heterodimers that bind to cis-acting elements. Its typical conserved motif [N-X(7)-R/K-X(9)-L-X(9)-L-X(6)-L-X(6)-L] may bind to specific DNA sequences [26] (Figure 1C).

Functional characterization in Arabidopsis confirmed that *DlAP1* acts as a typical class A AP1-like gene. Its overexpression promotes flowering in Arabidopsis and affects floral organ development, particularly in pistils and petals. Similar phenotypes were observed for other *AP1-like* genes (e.g., *GmAP1*, *DnAPL1*, *IiAP1*) when overexpressed in Arabidopsis [27,28,29]. In addition, overexpression of *DlAP1* in Arabidopsis results in leaf curling (Figure 4B), which is manifested as a spoon-shaped cauline leaf, and has a similar function to that of Arabidopsis *FUL*, namely it affects leaf development [30]. It is noteworthy that in a previous study, *DlAP1*-1 and *DlAP1*-2, two longan homologs of *AP1*, were also overexpressed in Arabidopsis, but no significant phenotypes were observed compared with Col-0 [13]. We attributed this phenotypic difference to sequence variations between the longan cultivars used. In this study, we used *D*. *longan* cv. ‘Shixia’, a premium cultivar widely cultivated in mainland China, Hong Kong, and Macao, while the comparative study utilized *D. longan* cv. ‘E-Daw’ in Thailand. In the sequence comparison, there were 10 and 13 amino acid differences in *DlAP1* with *DlAP1*-1 and *DlAP1*-2, respectively (Figure 1A), and perhaps differences in the key regions (i.e., the MADS-box, the K-box, and the C-terminal ‘CFAA’ base sequences) led to the phenotypic differences.

In contrast, *DlFD* overexpression cannot cause obvious phenotype in flowering time, which is the same as that of other plants (e.g., *OsFD1*, *ZmFDP*, *PtFD*, *SlFD*) in Arabidopsis [31,32,33,34]. We speculate that it may be due to the dependence of *DlFD* on species-specific interaction partners to activate the downstream pathway to function, such as protein OsFD1 dependence on Hd3a and 14-3-3 proteins; or similarly with the interaction network of maize ZmFDP and FD, there may be differences, and FD may have a different interaction network. The regulatory mechanism of *DlFD* deserves to be further elucidated in the future.

Subcellular localization showed that DlFT1 and DlFT2 localize to the nucleus and membrane, similar to other FT homologs (e.g., OsHd3a, SP5G, CiFT) [25,35,36,37], while DlAP1 and DlFD are exclusively nuclear, consistent with their roles as transcription factors and homologs (IiAP1, PtFD) [2,20,23,38]. These conserved localization supports their potential for physical interaction in vivo. Finally, our protein–protein interaction assays confirmed that both DlFT1 and DlFT2 interact with DlFD, whereas DlAP1 binds specifically to DlFT1. Although DlFT1 and DlFT2 share high sequence similarity and conserve key FT functional sites Tyr84 and Gln139, they differ significantly in DlAP1 binding. The functional difference between DlFT1 and DlFT2 may result from key structural variations. First, DlFT1 maintains conserved P-loop residues (Tyr134/Gln137/Trp138) while DlFT2 changed Tyr133 to Asn133. Second, DlFT1 retains an intact C-terminal domain (KDFAE/LYN) [13], whereas DlFT2’s altered structure likely affects DlAP1 binding, potentially explaining their opposing roles in flowering regulation.

However, we also noted notable interaction specificity: in the reciprocal assay, longan DlAP1 failed to bind to Arabidopsis AtFT. This asymmetric pattern suggests that the direct FT–AP1 interaction may exhibit species-specific constraints, though the structural basis for this selectivity remains unclear. At the same time, we cannot exclude the possibility that this observation stems from technical limitations of the Y2H system. For example, heterologous expressed proteins may not attain native folding, post-translational modifications, or proper subcellular localization in this experimental context. Therefore, we consider these Y2H results for DlAP1-AtFT as preliminary evidence for direct protein–protein interactions, rather than conclusive proof.

## 4. Plant Materials and Methods

### 4.1. Plant Material and Culture Conditions

For the natural flowering, the leaves and bud tissues of longan cultivar ‘Shixia’ were collected from the National Fruit Tree Germplasm Longan and Loquat Accessions Nursery, Fuzhou, China. Three trees, each over 10 years old in stable flowering phase, and well-managed in terms of fertilization and pest control were selected as biological replicates. Sampling was conducted every 10 days beginning on January 15, continuing until floral bud differentiation reached the “small red dot” stage. In total, six sampling points were collected: 50, 40, 30, 20, 10, and 0 days before floral bud morphological differentiation (DBF).

For the off-season flowering experiment, KClO_3_-induced floral induction was carried out in Dongfang City, Hainan Province, China, on 20 September 2023. The trees’ growth state and management were the same as the trees of natural flowering. Due to the rapid floral induction response, bud and leaf tissues were collected at three time points: 25, 15, and 0 DBF.

Heterologous gene overexpression was performed using Columbia-0 Arabidopsis (Col-0). Seeds were water-soaked and placed at 4 °C for 3 days, then sterilized (alcohol for 1 min, 10% NaClO for 10 min) and sown on 1/2 MS medium. After 3 days of dark treatment, the plates were transferred to normal culture conditions (22 ± 1 °C, 16-h light/8-h dark cycle). Seedlings were transplanted into soil (substrate:vermiculite = 3:1) 7 days after sowing. Subcellular localization and BiFC experiments were carried out using Ben’s tobacco (*Nicotiana benthamiana*) under culture conditions of 30 ± 1 °C, 16 h light/8 h darkness.

### 4.2. Gene Cloning

Gene cloning was performed using the E.Z.N.A. Total RNA Kit I (Omega, Biel/Bienne, Switzerland) to extract the total RNA of longan, and the NovoScript^®^ II Reverse Transcriptase (novoprotein, Suzhou, China) Kit was used to reverse transcribe the total RNA and remove the gDNA to generate cDNA. The coding sequences (CDS) of *DlAP1* and *DlFD* were used as queries to identify homologous sequences via local BLAST (version: 2.15.0; e-value cutoff: 1 × 10^−3^) in the local genome file to find one homologous sequence each, named *DlAP1*, *DlFD*. This was based on the sequence information to design primers, cDNA as a template for PCR, cloning to obtain CDS sequences, and then sequencing.

### 4.3. Sequence Analysis and Evolutionary Tree Construction

We retrieved homologous protein sequences from the following databases: NCBI (https://www.ncbi.nlm.nih.gov/ accessed on 30 June 2025) [39], TAIR (https://www.arabidopsis.org/ accessed on 30 June 2025) [40], SapBase (http://www.sapindaceae.com accessed on 30 June 2025) [17], and China Rice Data Center (www.ricedata.com accessed on 30 June 2025) for homologous protein sequence search and acquisition. The sequences were searched and obtained using MEGA7.0 [41] and GeneDoc2.7 for sequence comparison; phylogenetic trees were constructed using the neighbor joining method of MEGA7.0 and ChiPlot (https://chiplot.online/ accessed on 30 June 2025).

### 4.4. Heterologous Overexpression and Phenotypic Observation of Arabidopsis

The *pMDC202-DlAP1* and *pMDC202-DlFD* overexpression constructs were generated via homologous recombination by ligating the CDS sequences of *DlAP1* and *DlFD* into the *pMDC202* vector. After the vectors were sequenced correctly, they were transformed into Agrobacterium GV3101, and Col-0 plants were transformed by the Agrobacterium-mediated pollen tube pathway [42]. T1 generation plants were initially screened using 1/2MS+Hygromycin B (30 mg L^−1^) medium, then the total DNA from leaves of T1 generation plants was extracted for PCR to determine the positive seedlings, and T2 generation plants were screened for the 3:1 segregation ratio of the T1 generation, which were considered to be single-insertion plants. If all the seeds of the T3 generation plants were sown on the medium and all of them were well-grown without any segregation of the traits, they were considered to be pure hybrids and could be used for phenotypic observation.

Flowering time was accessed by recording the timing of inflorescence axis emergence and the number of rosette leaves at bolting (only rosette leaves bearing trichomes was counted). Additionally, leaf morphology, the number and morphology of flowering organs, and their modes of attachment were examined in 30 individual T3 generation plants, with a total of more than 50 flowers observed.

### 4.5. Gene Expression Level Detection by RT-qPCR

Total RNA was extracted from longan bud and leaf tissues using the RNA extraction Kit RC401 (Vazyme, Nanjing, China). RNA concentration and integrity were assessed using a Nanodrop spectrophotometer and agarose gel electrophoresis. For each sample, 1 µg of total RNA was reverse-transcribed into first-strand cDNA using the cDNA synthesis kit R412-01 (Vazyme, Nanjing, China).

*DlACTIN3* was used as the internal reference gene. Gene specific primers of five longan genes were designed using Primer v5 (Table S1). RT-qPCR was performed in a 20 µL reaction containing 1 μL cDNA (200 ng μL^−1^), 0.4 μL of each 10 μmol L^−1^ upstream and downstream primers, 10 μL SYBR Green reagent Q713-02 (Vazyme, Nanjing, China), and ddH_2_O μL to a final volume of 20 μL. Reactions were carried out on a Bio-Rad CFX-96 real-time PCR system (Bio-Rad, Hercules, CA, USA) with the following program: initial denaturation at 95 °C for 30 s, followed by 40 cycles of 95 °C for 10 s, annealing at 60 °C for 30 s. A melt curve analysis was performed to confirm amplification specificity.

Relative expression levels were calculated using the 2^−ΔΔCt^ method. Each reaction included three biological replicates and two technical replicates. Data are presented as mean ± SE, and statistical significance was determined by ANOVA followed by Tukey’s multiple comparisons test using R v4.4.1.

### 4.6. Subcellular Localization

The subcellular localization vector *p2300-GFP-DlFT1*, *p2300-GFP-DlFT2*, *p2300-GFP-DlAP1*, and *p2300-GFP-DlFD* recombinant vector were constructed by homologous recombination by ligating *DlFT1*, *DlFT2*, *DlAP1* and *DlFD* with the subcellular localization vector *p2300-GFP* (abbreviated as *GFP-DlFT1*, *GFP-DlFT2*, *GFP-DlAP1*, *GFP-DlFD*), and the empty *p2300-GFP* vector as control (abbreviated as *GFP*). The vectors were transformed into Agrobacterium GV3101 and correct sequencing. The successfully transformed Agrobacterium was cultured to a cell concentration = 0.6–0.8 (OD_600_), and then the OD_600_ was adjusted to 1.0 with the infiltration solution (100 mL of infiltration solution: 2 mL of 500 mM MES + 0.15 mL of 100 mM acetobutyrophenone + 10 mL of 100 mM MgCl_2_), and then placed in darkness at 28 °C for 2 h. Then, 3-week-old leaves of Ben’s tobacco were injected, and the leaves were incubated in darkness for 24 h, followed by normal light for 12 h. The leaves were incubated in darkness for 24 h, and then for 12 h with DAIP dye (4.4 mM MES). The leaves were stained with DAIP (4′,6-diamidino-2-phenylindole, dihydrochloride) as the nuclear maker and PI (propidium iodide) dye as the membrane maker, and were observed and photographed under a white laser light sheet microscope (Leica Microsystems, Wetzlar, Germany) for observation and photography.

### 4.7. Yeast Two-Hybrid

Using the GAL4 system, *pGBKT7-DlFT1*, *pGBKT7-DlFT2* recombinant vectors were constructed by ligating *DlFT1*, *DlFT2* and the bait vector *pGBKT7* using homologous recombination; *DlAP1*, *DlFD* and the prey vector *pGADT7* were ligated to construct the *pGBKT7-DlFT1*, *pGBKT7-DlFT2* recombinant vector. The *pGADT7-T + pGBKT7-lam* was used as the positive control, and *pGADT7-T*, *pGBKT7-53* was used as the positive control, as shown in the figure to combine the 8 groups of vectors, then co-transformed into Y2H yeast susceptible, plated on 2D synthetic dropout medium (-Trp/Leu), and cultured at 30 °C for three days. Next, monoclonal colonies were transferred to the 4D synthetic dropout medium (-Trp/-Leu/-Ade/-His) with 20 mM 3-amino-1,2,4-triazole (3-AT, Coolaber) and cultured at 30 °C for 4 days.

### 4.8. Bimolecular Fluorescent Complementary

The *pSPYNE-35S-DlFT1*, *pSPYNE-35S-DlFT2* recombinant vectors (referred to as *YNE-DlFT1*, *YNE-DlFT2*) and *pSPYEC-35S-DlAP1*, *pSPYEC-35S*-*DlFD* recombinant vectors (abbreviated as *YCE-DlFT1*, *YCE-DlFT2*) were constructed by ligating *DlAP1*, *DlFD*, and *pSPYCE-35S* vectors. Each of the 8 vector combinations (as shown in the figure) was transformed into *Agrobacterium tumefaciens* GV3101, 1:1 mixture of transient transformation of Ben’s tobacco; the transformation and cultivation methods were the same as in 4.5, and the same in the white laser light sheet microscope (Leica TCS SP8X DLS) under the observation of the photographs.

### 4.9. Data Statistics and Analysis

Data analysis was performed using GraphPad Prism9.5 (GraphPad Software, San Diego, CA, USA). Two-way ANOVA was employed to assess differences among groups, followed by Tukey–Kramer for multiple comparisons. Results with *p* < 0.01 were considered highly significant, those with *p* < 0.05 were deemed statistically significant, and differences with *p* < 0.1 were regarded as showing a trend.

## 5. Conclusions

In this study, two key flowering-related genes were identified and resolved in longan: *DlAP1* (*euAP1* gene of the AP1/SQUA subfamily) and *DlFD* (bZIP family transcription factor). Analysis of the gene structure and expression pattern showed that *DlAP1*, although it has the function of *AP1*, is different from the interaction between AP1 and FT. Its functional studies showed that *DlAP1* not only participates in the regulation of floral organ development, but also directly interacts with *DlFT1* in the nucleus, thus forming a key functional module in the flowering regulatory network, suggesting its unique role in longan flowering. In addition, we confirmed the nuclear localization properties of proteins DlAP1 and DlFD as well as the dual localization of DlFT1/2 in the nucleus and membrane. DlFD physically interacts with DlFT1/2 in the nucleus, suggesting that it may synergistically mediate florigen signaling. These results reveal the role of DlAP1 as a core node for integration of flowering signaling in longan, linking the conserved FT pathway to species-specific developmental adaptations. This study provides a new basis for in-depth analysis of the molecular mechanism of longan flowering and lays the foundation for subsequent exploration of ‘DlAP1–DlFT1’ regulation.

## Data Availability

The data presented in this study are available on reasonable request from the corresponding author.

## Supplementary Materials

The following supporting information can be downloaded at: https://www.mdpi.com/article/10.3390/plants15010106/s1, Figure S1: Yeast two-hybrid analysis of interactions between AtAP1 and DlFT1, DlAP1and AtFT, AtAP1 and AtFT; File S1: The CDS sequences of DlAP1, DlFD, DlFT1, and DlFT2 genes in longan (*Dimocarpus longan*); Table S1: Primers used for RACE experiments, construction of vector and RT-qPCR.
