# Supplementary material for: Functional Characterization of Floral Gene Network Reveals a Critical FT1–AP1 Interaction in Flowering Regulation in Longan
Source: Plants (Basel). 2025 Dec 30;15(1):106. doi: 10.3390/plants15010106 (PMC12787483; doi:10.3390/plants15010106)
Supplement: Supplementary file 1 [file plants-15-00106-s001.zip › plants-4064820-supplementary.pdf]

Functional Characterization of Floral Gene Network Reveals a Critical FT1–AP1 Interaction in Flowering Regulation in Longan

Yuru Tang [1,2,†], Yating Xu [1,2,†], Haoming Mao [1,2], Yawen Xu [1,2], Jianling Pan [1,2], Shaoquan Zheng [3], Guochun Zhao [1,2,*], Wenshun Hu [3,*] and Ray Ming [1,2,*]

[1] Fujian Provincial Key Laboratory of Haixia Applied Plant Systems Biology, Center for Genomics and Biotechnology, Fujian Agriculture and Forestry University, Fuzhou 350002, China

[2] Key Laboratory of Genetics, Breeding and Multiple Utilization of Corps, Ministry of Education, Fujian Agriculture and Forestry University, Fuzhou 350002, China

[3] Fujian Breeding Engineering Technology Research Center for Longan and Loquat, Fruit Research Institute, Fujian Academy of Agricultural Sciences, Fuzhou 350013, China

* Correspondence: zhaoguochun1122@126.com (G.Z.); huwenshun06@163.com (W.H.); rayming@illinois.edu (R.M.)

† These authors contributed equally to this work.

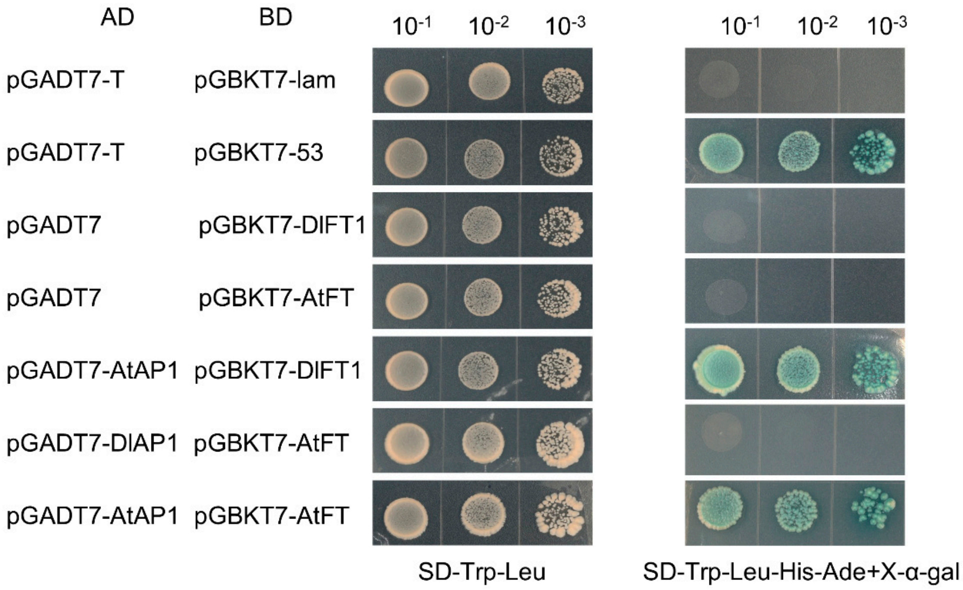

Figure S1. Yeast two-hybrid analysis of interactions between AtAP1 and DlFT1, DlAP1and AtFT , AtAP1 and AtFT.

File S1. The CDS sequences of DlAP1, DlFD, DlFT1, and DlFT2 genes in longan (*Dimocarpus longan*)

>DlAP1
ATGGGGAGAGGTAGGGTTCAGTTGAAGAGGATAGAGAACAAGATCAATCGCCAGGTGACGTTTTCCAA
AAGGAGAGCTGGTTTGTTGAAGAAAGCTCATGAGATCTCTGTGTTGTGTGATGCTGAGGTTGCTCTGATT
GTCTTCTCTCACAAAGGGAAGCTCTTTGAGTATGCCACTGATTCTAGCATGGAAAAGATCCTGGAACGC
TATGAGAGGCACAATTACACAGAGAGGCGGCTGGTTGCAACTGATCCTGAATCACAGGTGAACTGGGC
CATGGAGTATAACAGACTTAAGGCCAAAATTGAGCTTCTACAAAGAAACCACAGGCACTACATGGGAG
AAGATTTAGACTCTCTGAGTCTAAAAGAGATCCAAAATTTGGAACAGCAGCTTGACACTTCACTTAAGC
ACATTAGATCCAGAAAGAACCAACTGATGTATGATTCCATCTCTGAGCTTCAAAGAAAGGAAAAGGCA
ATACTGGAGCAAAACAATATGCTAGCAAAGCAGATCAAGGAGAAGGAGAAAACTATGGCTCAACAAGT
GCAGTGGGAGCAGCAAAATCAGGGCCCAAATACACTCTCCTTCCTGGTCCCAATGCCACCTCCCTGCTT
AAAAATGGGTGGCATTTATCAGGAAGAAGCTCCAGAGATAAGGAGGAACGAGCTTGACCTCACTCTTG
AACCCTTATATTCGTGCAATCTTGGATGTTTCGCTACATGA

>DlFD
ATGGATGAAGACGATGATACCACCTATATTAACAACACCTCCTCCGCCTGCACCATCAGCTCCATCACTC
TGATTAATAATAACATGAACGGTCCTGATCCTCCTCCTCCTCATCGTCATCAGTATCAGCAACATCT
CCCATTTCAATCAGCATCCTCTACTTCCTATAGTGTCACACATACTACTATAAGTCACAGCAAACCAATT
AACATGGAAGAAGTGTGGAATGACCTCAACTTGGTTCCTCTAAACGAGGGTTTGTCTTTGAACAACAAC
AGCAAAACCCCACCAAACACATCCACTGTTTTTGGGGTTCATGACTTTCTAGGGAGATCTAATGATACTA
GGAATCTTCAGTGTAGTAGCAATATTGGAGAGATAAATAGGTCTGTTTTTTGTTCTCATGATAGTCAAAT
GCAACAACAGCAACAATTATCTCTTGGGCCTGCTTTGAACCTGAACTCTAGCCCTGGTCTTGAGTTCTTG
GTAGACAACTTTGATCCACTGGTCATTTCAAGCAACTCTGATCAGATCATGATCCAACCAAACCCCATTT
CGAGTGTTGGTCGTGTGCCTCCACATGAGGATCATCGACTCACGAACGATGATTACGGTAGCCCTTTTCA
GGCCTTGGCTTCTGCTACTCGGTTGAATACTGTTGGCAAGAGGCGGCTCCATGAAAATGTAGCTGAGAG
GCGTCGCAAGCGTAGGATCAAGAACCGCGAGTCTGCTGCTCGATCTAGGGCTAGAAGAGAGGCATATA
CCAACGAATTGGAGATGGAACTTAGTCATCTGCTATCTGAAAATTCAAAGCTTAAAAAGCAACATGAAC
AGTTGCGCTTGGTTGTGGACGCTCAAGTTGCCGGGAGGCATGTGCTTCACCGGGCGTCAACTGCTCCATT
TTGA

>DlFT1
ATGCCTCGAGATAGAGATCCTCTTGTGGTTGGCCGAGTGATAGGGGATGTTCTTAATCCTTTCACAAAGT
CAATTTCTCTTAGTGTTTCCTATAACAACAGGGAAATCAGCAATGGTTGTGAGCTCAAACCCTCTCAGAT
TGTCAACCAACCTAGGGTTGATATTGGTGGTGATGATCTCAGGACCTTCTACACTTTGGTCATGGTGGAT
CCTGATGCACCCAGCCCAAGTGAACCAAGACTGAGGGAGTACTTGCATTGGTTGGTAACTGATATTCCA
GCGACCACTGGGGCAACCTTTGGGCAAGAGGTTGTGTGTTATGAGAGCCCTAGACCAACATCGGGGATT
CATAGGTTCATATTCGTCTTGTTCCGGCAACTGGGCAGGCAGACAGTGTACGCGCCGGGATGGCGCCAG
AATTTCAACACCAAAGAATTTGCTGAGCTTTACAACCTTGGATCTCCGGTTGCCGCGGTCTACTTTAACA
GCCAGAGAGAGAGTGGCTCCGGTGGAAGGAGACGATAA

>DlFT2
ATGTCTAGAAATAGAGATCCTCTTGCGGTTGGCCGAGTGATAGGGGATGTTCTTGATCCCTTCACAAGGT
CAATTTCTCTAAGTATTTCCTATAACAACAGGGCAATCAACAATGGTTATGAGCTCAAACCCTCTCAGAC
TGTTAACCAACCTAGGGTTGATATTGGTGGTGATGATCTCAGGACCTTCTACACTTTGGTCATGGTGGAT
CCTGATGCACCGAGCCCGAGTGACCCAACCCTGAGAGAGTACTTGCATTGGCTGGTGACAGACATCCCC
GCGACCACTGGGGCAACCTTTGGGCAAGAGGCTGTGAGTTATGAGAGCCCCAGACCAACTGTGGGGATT
CACAGGTTCGTATTCGTGTTGTTCCGGCAACAAGGCAGGCAGACAGTGAATGCACCTGGCTGGCGACAG
AATTTCAAGACCAAAGACTTTGCTGAGCTTTACAACCTTGGATCACCGGTGGCCGCTGTCTACTTCAACT
GCCAGAGGGAGATTATCTCTGGTGGAAGCAGACGATAA

Table S1. Primers used for RACE experiments, construction of vector and RT-qPCR.

Name	Sequence (5′→3′)	Note
5′-DlAP1	ATGGGGAGAGGTAGGGTTCA	5′-RACE
3′-DlAP1	TCATGTAGCGAAACATCCAAGA	3′-RACE
5′-DlFD	ATGGATGAAGACGATGATACCAC	5′-RACE
3′-DlFD	TCAAAATGGAGCAGTTGACGC	3′-RACE
5′-DlFT1	ATGCCTCGAGATAGAGATCCTCTT	5′-RACE
3′-DlFT1	TTATCGTCTCCTTCCACCGGA	3′-RACE
5′-DlFT2	ATGTCTAGAAATAGAGATCCTCTTGCG	5′-RACE
3′-DlFT2	TTATCGTCTGCTTCCACCAGAGAT	3′-RACE
DlAP1-OE-F	GAGGACCTCGACTCTAGAATGGGGAGAGGTAGGGTTCA	Overexpression vector
DlAP1-OE-R	CATTTTTTCTACCGGTACCTCATGTAGCGAAACATCCAAGA	Overexpression vector
DlFD-OE-F	GAGGACCTCGACTCTAGAATGGATGAAGACGATGATACCAC	Overexpression vector
DlFD-OE-R	CATTTTTTCTACCGGTACCTCAAAATGGAGCAGTTGACGC	Overexpression vector
DlFT1-GFP-F	GGGCCCGGGGTCGACATGCCTCGAGATAGAGATCCTCTT	Subcellular localization vector
DlFT1-GFP-R	TACCGGATCCACTAGTTCGTCTCCTTCCACCGGA	Subcellular localization vector
DlFT2-GFP-F	GGGCCCGGGGTCGACATGTCTAGAAATAGAGATCCTCTTGCG	Subcellular localization vector
DlFT2-GFP-R	TACCGGATCCACTAGTTCGTCTGCTTCCACCAGAGAT	Subcellular localization vector
DlAP1-GFP-F	GGGCCCGGGGTCGACATGGGGAGAGGTAGGGTTCA	Subcellular localization vector
DlAP1-GFP-R	TACCGGATCCACTAGTTGTAGCGAAACATCCAAGA	Subcellular localization vector
DlFD-GFP-F	GGGCCCGGGGTCGACATGGATGAAGACGATGATACCAC	Subcellular localization vector
DlFD-GFP-R	TACCGGATCCACTAGTAAATGGAGCAGTTGACGC	Subcellular localization vector
DlFT1-Y2H-F	CATATGGCCATGGAGGCCGAATTCATGCCTCGAGATAGAGATCCTCTT	Yeast two-hybrid vector
DlFT1-Y2H-R	TGCGGCCGCTGCAGGTCGACTTATCGTCTCCTTCCACCGGA	Yeast two-hybrid vector
DlFT2-Y2H-F	CATATGGCCATGGAGGCCGAATTCATGTCTAGAAATAGAGATCCTCTTGCG	Yeast two-hybrid vector
DlFT2-Y2H-R	TGCGGCCGCTGCAGGTCGACTTATCGTCTGCTTCCACCAGAGAT	Yeast two-hybrid vector
DlAP1-Y2H-F	GCCATGGAGGCCAGTGAATTCATGGGGAGAGGTAGGGTTCA	Yeast two-hybrid vector
DlAP1-Y2H-R	TGCAGCTCGAGCTCGATGGATCCCTCATGTAGCGAAACATCCAAGA	Yeast two-hybrid vector
DlFD-Y2H-F	GCCATGGAGGCCAGTGAATTCATGGATGAAGACGAT	Yeast two-hybrid vector

	GATACCAC	
DlFD-Y2H-R	TGCAGCTCGAGCTCGATGGATCCCTCAAAATGGAGC AGTTGACGC	Yeast two-hybrid vector
DlFT1-BiFC-F	CGCCACTAGTGGATCCATGCCTCGAGATAGAGATCC TCTT	BiFC vector
DlFT1-BiFC-R	TACCCCTCGAGGTCGACTCGTCTCCTTCCACCGGA	BiFC vector
DlFT2-BiFC-F	CGCCACTAGTGGATCCATGTCTAGAAATAGAGATCC TCTTGCG	BiFC vector
DlFT2-BiFC-R	TACCCCTCGAGGTCGACTCGTCTGCTTCCACCAGAG AT	BiFC vector
DlAP1-BiFC-F	CGCCACTAGTGGATCCATGGGGAGAGGTAGGGTTCA	BiFC vector
DlAP1-BiFC-R	TACCCCTCGAGGTCGACTGTAGCGAAACATCCAAGA	BiFC vector
DlFD-BiFC-F	CGCCACTAGTGGATCCATGGATGAAGACGATGATAC CAC	BiFC vector
DlFD-BiFC-R	TACCCCTCGAGGTCGACAAATGGAGCAGTTGACGC	BiFC vector
ACTIN2-F	TCCCTCAGCACATTCCAGCAGAT	AtActin
ACTIN2-R	AACGATTCCTGGACCTGCCTCATC	AtActin
DlACTIN3-F	TCAGGCAGTCCTCTCTCTTTA	DlActin
DlACTIN3-F	ATAGCATGAGGAAGGGCATATC	DlActin
DlFT1-QRT-F	AGTTGCCGGAACAAGACGAA	RT-qPCR
DlFT1-QRT-R	TGGGCAAGAGGTTGTGTGTT	RT-qPCR
DlFT2-QRT-F	TTGGCTGGTGACAGACATCC	RT-qPCR
DlFT2-QRT-R	CGAACCTGTGAATCCCCACA	RT-qPCR
DlAP1-QRT-F	GCAGTGGGAGCAGCAAAATC	RT-qPCR
DlAP1-QRT-R	TAAGCAGGGAGGTGGCATTG	RT-qPCR
DlFD-QRT-F	AAGAGGCGGCTCCATGAAAA	RT-qPCR
DlFD-QRT-R	TCGCGGTTCTTGATCCTACG	RT-qPCR
